# Antihypertensive effects of the hydro-ethanol extract of *Senecio serratuloides* DC in rats

**DOI:** 10.1186/s12906-019-2463-2

**Published:** 2019-02-28

**Authors:** Charlotte Mungho Tata, Constance Rufaro Sewani-Rusike, Opeoluwa Oyehan Oyedeji, Ephraim Tobela Gwebu, Fikile Mahlakata, Benedicta Ngwenchi Nkeh-Chungag

**Affiliations:** 1 Department of Human Biology, Faculty of Health Sciences, Walter Sisulu University, Mthatha, 5117 South Africa; 2 Department of Chemistry, Faculty of Science and Agriculture, University of Fort Hare, PBX1314, Alice, Eastern Cape Province 5700 South Africa; 3 Department of Chemistry, Faculty of Science and Technology, Rusangu University, Monze, Zambia; 4 Traditional Healer, Lusikisiki, Eastern Cape South Africa; 5 Department of Biological Sciences, Faculty of Natural Sciences, Walter Sisulu University, Mthatha, 5117 South Africa

**Keywords:** *Senecio serratuloides*, N-nitro-L-arginine methyl ester, Hypertension, Lipid profile

## Abstract

**Background:**

*Senecio serratuloides* DC is used in folk medicine for treating hypertension, skin disorders, internal and external sores, rashes, burns and wounds. This study aimed at investigating the antihypertensive effects of the hydroethanol extract of *S. serratuloides* (HESS) in N-Nitro-L-arginine methyl ester (L-NAME) induced hypertension in rats.

Methods: Acute toxicity of HESS was first determined to provide guidance on doses to be used in this study. Lorke’s method was used to determine safety of the extract in mice. Female Wistar rats were treated orally once daily with L-NAME (40 mg/kg) for 4 weeks and then concomitantly with L-NAME (20 mg/kg) and plant extract (150 and 300 mg/kg), captopril (20 mg/kg) or saline as per assigned group for 2 weeks followed by a 2-week period of assigned treatments only. Blood pressure was monitored weekly. Lipid profile, nitric oxide, renin and angiotensin II concentrations were determined in serum while mineralocorticoid receptor concentration was quantified in the kidney homogenate. Nitric oxide (NO) concentration was determined in serum and cardiac histology performed.

**Results:**

HESS was found to be non-toxic, having a LD_50_ greater than 5000 mg/kg. Blood pressure increased progressively in all animals from the second week of L-NAME treatment. HESS treatment significantly and dose-dependently lowered systolic blood pressure (*p* < 0.001), diastolic blood pressure (*p* < 0.01), low density lipoprotein cholesterol (*p* < 0.01) and triglycerides (*p* < 0.01). It significantly prevented L-NAME induced decrease in serum angiotensin II (*p* < 0.01), high density lipoprotein cholesterol (*p* < 0.001) and serum nitric oxide concentrations (*p* < 0.001). HESS also significantly (*p* < 0.01) prevented collagen deposition in cardiac tissue.

**Conclusion:**

The hydro-ethanol extract of *Senecio serratuloides* showed antihypertensive, antihyperlipidemic and cardioprotective effects in rats thus confirming its usefulness in traditional antihypertensive therapy and potential for antihypertensive drug development.

## Background

One in three adults worldwide has hypertension (HTN) and the prevalence has been shown to increase with age [1]. Hypertension is a growing public health concern in sub-Saharan countries where it was previously not reported. In this population, HTN is characterized by a rapid onset, poor control and an early onset of target organ damage [2]. Poor control of HTN contributes enormously to the burden of cardiovascular diseases (CVDs) and associated morbidity and mortality. Indeed, a relevant systemic meta-analysis showed that every 10 mmHg reduction of systolic blood pressure (BP) was accompanied by a significant decrease in the risk for CVD [3]. Several classes of anti-hypertensive medications have been developed with the aim of reducing BP and consequently the associated risks [4]. However, affordability and availability of pharmaceutical antihypertensive medications are important challenges especially in rural African communities thus affecting compliance with treatment regiments. Importantly, reported side effects of pharmaceutical drugs [5] and the belief that plant medicines are less toxic are contributing to the preference of plant extracts over pharmaceutical drugs [6].

In rural South African communities, medicinal plants are used for BP management. *S. serratuloides* DC, is an Asteraceae commonly found in areas of South Africa which receive summer rainfalls. *S. serratuloides* is a perennial which grows from a woody root/stem, has serrated leaves and tiny yellow flowers which are clustered at the terminals. It is used in folk medicine for treatment of various ailments [7, 8]. A recent ethnobotanical survey of medicinal plants used for self-medication by lay people of Maputaland and Northern Kwazulu-Natal, South Africa, showed that *S. serratuloides* was one of the most commonly used plants for treatment of HTN [9, 10]. Furthermore, *S. serratuloides* was reportedly used in combination with several medicinal plants to prepare concoctions for hypertension treatment [11]. Importantly none of the plant combinations which included *S. serratuloides* had been previously described. Other authors from the same region indicated that *S. serratuloides* was among the most used plants for respiratory infections [12] thus confirming the fact that this plant has extensive uses. Although *S. serratuloides* is widely used in the South African traditional medicine, it’s medicinal properties have not received much scientific attention. Extracts of this plant are reported to have antioxidant, analgesic, anti-inflammatory and wound healing activities [13, 14]. Due to the efficacy of the plant extract in treating various diseases it is traditionally referred to as the ‘*two day cure*’ plant. Although there is no scientific report on the use of *S. serratuloides* in the treatment of hypertension, another member of the Genus, *Senecio nutans* which is native to South America has demonstrated antihypertensive and hypotensive effects in rats [15]. The health benefits of the Senecios may be associated with their rich flavonoid and phenol contents [16]. Indeed, studies have demonstrated the usefulness of plant flavonoids in the prevention of atherosclerosis and hyperlipidemia [17] though these properties have not been evaluated for *S. serratuloides*.

Even though *S. serratuloides* is popular in South African traditional medicine, only its antimicrobial, analgesic and anti-inflammatory/wound healing properties have been investigated [13, 14]. Its potential usefulness in the management of any of the chronic diseases of lifestyle has not been studied. To our knowledge, this is will be the first study reporting on the antihypertensive effects of *S. serratuloides*. In this study we used L-NAME to induce hypertension. L-NAME is an inhibitor of nitric oxide synthase activity and consequently the synthesis of nitric oxide (NO). Chronic administration of L-NAME results in generalized reduction of NO resulting in endothelial dysfunction which is observed in the early phase of hypertension [18]. L-NAME induced hypertension is associated with activated sympathetic tone and generalized vasoconstriction [19]. This experimental model of hypertension has several similarities with human hypertension in the African population who demonstrate increased sympathetic activation, salt sensitivity and decreased NO dependent vasodilation [20] and high rate of target organ damage [21]. Therefore, the aim of the study was to investigate the safety and antihypertensive properties of the hydroethanol extract of *S. serratuloides* (HESS) in the L-NAME-induced hypertensive rat model and the effect of treatment on selected target organs.

## Methods

### Reagents

Triglyceride reagent (TR212), Low density lipoprotein reagent (CH2656), High density lipoprotein reagent (CH2655) and Triglyceride reagent (TR210) (Randox Laboratories Ltd., UK); Renin ELISA kit (E-EL-R0030) and Angiotensin II ELISA kit (E-EL-R1430) (Elabscience, PRC); Bradford reagent, Fastcast acrylamide kit, Turbo transfer kit (170–4270) and Clarity western ELC substrate (170–5060) (Bio-Rad Laboratories, USA); Anti-mineralocorticoid receptor antibody (ab2774), anti-ß-actin antibody (ab8227) and Goat anti-rat IgG H&L (HRP) (ab205719) (Abcam Laboratories Inc., USA) Protease inhibitor (S8820-20TAB), RIPA buffer (R0278), Nω-Nitro-L-arginine methyl ester and ß-mercaptoethanol (Sigma, USA); stains (Harris haematoxylin, eosin and picrosirius red) and solvents (ethanol, glacial acetic acid, methanol xylene) were of analytical grade.

### Plant material and extraction

*Senecio serratuloides* was supplied by Mr. Fikile Mahlakata, a traditional healer in Lusikisiki, South Africa and authenticated in the KEI Herbarium of Walter Sisulu University where a voucher specimen (Tata 1/13967) was deposited. Plant material was air dried in the laboratory, crushed and exhaustively extracted in 70% ethanol which extracts a good number of polar and non-polar secondary metabolites. Ethanol was recovered under vacuum using a rotary evaporator (Heidolph Laborota 4000, Germany) at 35 °C and then oven dried at the same temperature. The plant extract was then stored in a refrigerator and reconstituted in distilled water before use.

### Animals

Swiss albino female mice weighing 20–25 g were used for acute toxicity studies while female Wistar rats weighing 200–240 g were used for HTN study. Animals were procured from South African Vaccine Producers (Johannesburg, South Africa) and housed in cages in the Walter Sisulu University animal holding facility. Room temperature was maintained at 24 °C and natural light was used for lighting. Animals had free access to rat chow (Epol-SA) and tap water. All animal procedures were carried out in line with the South African National Standards: The care and use of animals for scientific purposes [22].

### Acute oral toxicity

Acute toxicity study was conducted in accordance with Lorke’s method as described by Bulus et al., [23]. The study was conducted in two phases using a total of sixteen mice. The geometric mean of the least dose that killed an animal and the highest dose that did not kill any animal was considered as the median lethal dose (LD_50_):

$$ {LD}_{50}=\surd \left({D}_0{xD}_{100}\right). $$

Where D_0_ is the highest dose that caused no mortality and D_100_ is the lowest dose that caused mortality.

### Hypertension study design

Animals were randomized into five treatment groups of six animals per group (*n* = 6) as follows:

- NT – normotensive control.
- LN – L-NAME treatment only.
- CPT + LN – CPT (Captopril (20 mg/kg)).
- HESS150 + LN – HESS (150 mg/kg).
- HESS300 + LN – HESS (300 mg/kg).

**Fig. 1:**
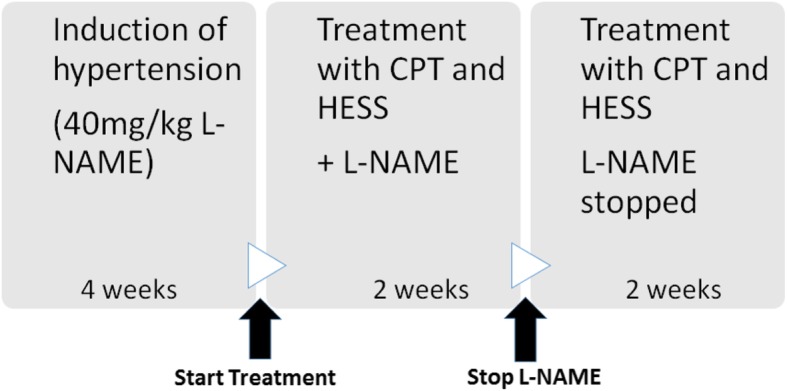
Timeline for induction of hypertension and treatment

Experiments were carried out according to the protocol described by Lane, [24] with slight modification. Except for the normotensive group that was treated with normal saline only, rats from the other groups were first treated with L-NAME (40 mg/kg) for 4 weeks and then the LN, CPT, HESS150 and HESS300 groups were co-treated with normal saline, captopril or extract (150 and 300 mg/kg) respectively and L-NAME (20 mg/kg) as per assigned groups for 2 weeks. Finally, in the last 2 weeks, LN treatment was discontinued for all groups, and the NT and LN groups were treated with normal saline, the CPT group with captopril and HESS groups with the extract. Summary of study timeline is shown in Fig. 1.

### Measurement of blood pressure

Blood pressure was measured in conscious rats, using non-invasive tail-cuff plethysmography (CODA™ 8 Non-Invasive Blood Pressure System, Kent Scientific Corporation, USA) as per manufacturer’s instructions. Systolic blood pressure (SBP), diastolic blood pressure (DBP) and heart rates (HR) were measured weekly at 8 am. Rats with SBP ≥ 200 mmHg and DBP ≥ 160 mmHg were considered hypertensive.

### Termination of treatment

Treatment was stopped 2 days before the termination in order to study the long-term effects of the extract without involvement of the effects of acute administration [25, 26]. Rats were fasted for 16 h, weighed and terminated individually by carbon dioxide inhalation followed by cardiac puncture for blood collection. Collected blood was placed in ST vacutainers, mixed and incubated in upright positions at room temperature for 45 min and centrifuged at 1300 RCF for 15 min to obtain serum. Kidneys and hearts were harvested and divided into two portions half of which was fixed in 10% buffered formalin and the other half stored at -20 °C for later analysis.

### Determination of lipid profile of treated animals

Triglycerides, low density lipoprotein cholesterol (LDL) and high density lipoprotein cholesterol (HDL), were measured using kits from Randox Laboratory (Randox co. UK) following protocol described by manufacturer. Total cholesterol and very low density lipoprotein (VLDL) were calculated using Friedewald equation as described by Vuilleumier et al., (2010) [27]:

VLDL = TG/5.

TC = HDL + LDL + VLDL.

### Determination of renin and angiotensin II concentration in serum

Renin and Ang II concentrations were determined in serum by enzyme linked immunosorbent assay (ELISA) using pre-coated commercial kits (Elabscience, PRC). Renin concentration was determined via sandwich-ELISA (E-EL-R0030) while angiotensin II concentration was determined by competitive ELISA (E-EL-R1430).

### Determination of mineralocorticoid receptor concentration in kidney homogenate

Mineralocorticoid receptors were separated and identified by western blotting using commercial kits (Bio-Rad laboratories, USA). Briefly, proteins in kidney homogenate were quantified and separated by electrophoresis and blotted onto a nitrocellulose membrane. The membrane was incubated with rat anti-mineralocorticoid antibody (ab2774) followed by incubation with horseradish peroxidase (HRP)-conjugated secondary antibodies; goat anti-rat IgG H&L (HRP) (ab205719) (Abcam, Laboratories Inc., USA) for 1 h at room temperature and 25 rev/min. Bound antibodies were detected by chemiluminiscence using Clarity Western enhanced chemiluminiscence (ECL) substrate (170–5060) and imaging was done using ChemiDoc Touch Imaging System (Bio-Rad laboratories, USA). Analysis of images was performed using Image Lab software (Bio-Rad laboratories, USA) and bands for mineralocorticoid receptors were normalized using housekeeping proteins (ß-actin, ab8227).

### Determination of NO levels in serum and tissue homogenates

Nitric oxide was quantified indirectly using Griess reagent (5% phosphoric acid, 1% N-(1-naphthyl) ethylenediamine (NEDD) and 1% sulfanilamide) and NaNO_2_ standard curve. In the assay, acidified NO_2_ produced a nitrosating agent which reacted with sulfanilic acid to produce diazonium ion which then coupled with NEDD to form a chromophoric azo-derivative which was quantified spectrophotometrically (Bio-Rad 680, USA) at 540 nm.

### Cardiac histology

Heart sections were fixed in 10% buffered formalin, embedded in paraffin wax, sectioned in 5-μm slices and stained with haematoxylin/eosin [28] and picrosirius red stain [29]. These sections were examined using a digital light microscope (Leica DMD108) at 20X and 40X magnifications and images captured. Morphometry was used to determine areas of cardiomyocyte thickening as previously described [30]. Semi-quantification of collagen was done using 100 μm × 100 μm images. Image J (NIH.gov/ij/) scientific image analysis software was used for density quantification as previously described [31]. Measurements were performed on 4 different slides per sample.

### Statistical analysis

Statistical analysis was carried out using GraphPad Prism version 5.03 for Windows (GraphPad Software, San Diego, CA, USA). One-way analysis of variance (ANOVA) followed by Tukey’s *posthoc* test for multiple comparisons was performed to determine differences between treatment groups. A *p-*value less than 0.05 was considered statistically significant. Results were expressed as mean ± standard error of the mean (SEM).

## Results

### Acute toxicity results

Animals did not show any signs of toxicity after treatment with HESS in line with the LD_50_ of HESS greater than 5000 mg/kg that was obtained. Wellness parameters such as sleep, behavioral pattern, skin, fur and appetite which are used for evaluation of toxicity were found to be normal up to a dose level of 5000 mg/kg during the entire period of observation. In addition, there was no relative difference, observed in the body weights of treated and control animals after two weeks of observation.

### Effect of HESS on systolic and diastolic blood pressures

**Fig. 2:**
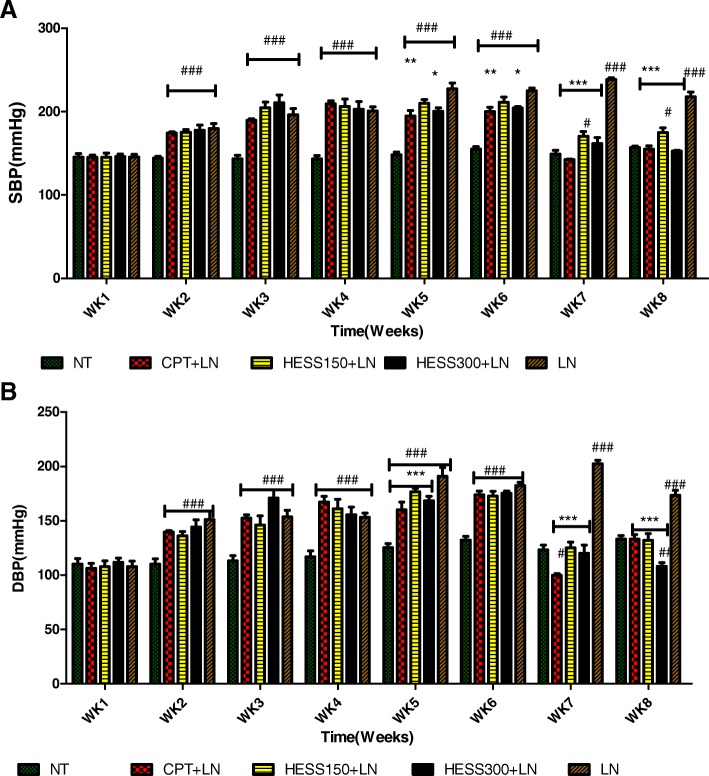
Effect of HESS on SBP (Panel **a**) and DBP (panel **b**) in L-NAME induced hypertension. Values are expressed as mean ± SEM. *n* = 6; NT = normotensive control; LN = L-NAME control; CPT + LN = captopril; HESS150 + LN and HESS300 + LN = hydroethanolic extract of *Senecio* serratuloides at 150 and 300 mg/kg respectively. * *p* < 0.05, ** p < 0.01, *** p < 0.001 compared to L-NAME (LN) control group; #*p* < 0.05, ## *p* < 0.01, ###*p* < 0.001 compared to normotensive control group

Both SBP and DBP increased significantly in all treatment groups compared to NT group from weeks 2–4 of L-NAME treatment. In the subsequent weeks, co-treatment with HESS and L-NAME for 2 weeks and treatment with HESS or normal saline only for 2 more weeks revealed that in weeks 5 and 6, CPT and HESS300 significantly decreased SBP by 7 and 1% respectively compared to 13% increase in LN group. In week 7, CPT, HESS150 and HESS300 significantly decreased SBP by 32, 17 and 20% compared to 7% increase in LN group. In week 8, CPT HESS150 and HESS300 significantly decreased SBP by 26, 15 and 25% compared to 0.1% decrease in LN group (Fig. 2, panel A). Considering the effect of treatment on DBP, it was observed that in week 7, CPT, HESS150 and HESS300 significantly decreased DBP by 40, 22 and 23% respectively compared to 16% increase in LN group. Meanwhile in week 8, CPT, HESS150 and HESS300 significantly decreased DBP by 20, 18 and 40% compared to 3% increase in LN group (Fig. 2, panel B).

### Lipid profile

Results from serum lipid profile showed that L-NAME significantly decreased HDL while increasing LDL, VLDL and TG in LN group compared to NT control group. HESS150 and HESS300 significantly increased HDL compared to LN control. However only HESS300 significantly decreased LDL, VLDL and TG (Table 1).

**Table 1:** Effect of HESS on lipid profile in L-NAME induced hypertensive rats

	Parameters (mg/dl)				
	TG	HDL	LDL	VLDL	TC
NT	77.4 ± 1	61.2 ± 5	46.61 ± 3	15.5 ± 0.2	121.3 ± 8
HESS150 + LN	86.5 ± 2^#^	50.99 ± 2**	57.98 ± 3^#^	17.3 ± 0.4^#^	124.9 ± 6
HESS300 + LN	69.2 ± 1^#^***	52.95 ± 3***	46.34 ± 4**	13.8 ± 0.3***^#^	116.6 ± 7
CPT + LN	79.5 ± 2	35.3 ± 4^###^	52.4 ± 4	15.9 ± 0.5	101.5 ± 8
LN	86.1 ± 2^#^	29.4 ± 2^###^	64.15 ± 2 ^##^	17.2 ± 0.4^#^	110.8 ± 5

Results are expressed as mean ± SEM, *n* = 6. NT = normotensive control group; LN = L-NAME control group; CPT + LN = captopril group; HESS150 + LN and HESS300 + LN = hydroethanolic extract of *Senecio* serratuloides at 150 and 300 mg/kg groups respectively. **p < 0.01, ***p < 0.001 compared to L-NAME (LN) control group; #*p* < 0.05, ##*p* < 0.01, ##*p* < 0.001 compared to normotensive control group

### Renin and angiotensin II concentration in serum

**Fig. 3:**
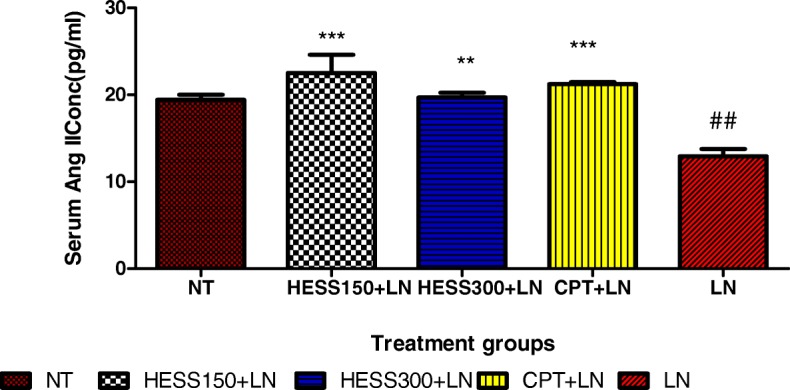
Serum Ang II concentrations. Values are expressed as mean ± SEM. n = 6; NT = normotensive control; LN = L-NAME control; CPT + LN = captopril; HESS150 + LN and HESS300 + LN = hydroethanolic extract of *Senecio serratuloides* at 150 and 300 mg/kg respectively. **p < 0.01, ***p < 0.001 compared to L-NAME (LN) control group; #*p* < 0.05, ##*p* < 0.01 compared to normotensive control group

There was no difference (*p* > 0.05) in concentration of renin between treatment groups (NT = 557 ± 32 pg/ml; LN = 480 ± 21 pg/ml; CPT + LN = 550 ± 19 pg/ml; HESS150 + LN = 561 ± 25 pg/ml; HESS300 + LN = 527 ± 29 pg/ml). L-NAME significantly decreased Ang II concentration in LN group compared to NT group. However, HESS150 and HESS300 significantly prevented this L-NAME-induced decrease in Ang II compared to LN group (Fig. 3).

### Mineralocorticoid receptor concentration in kidney tissue homogenate

**Fig. 4:**
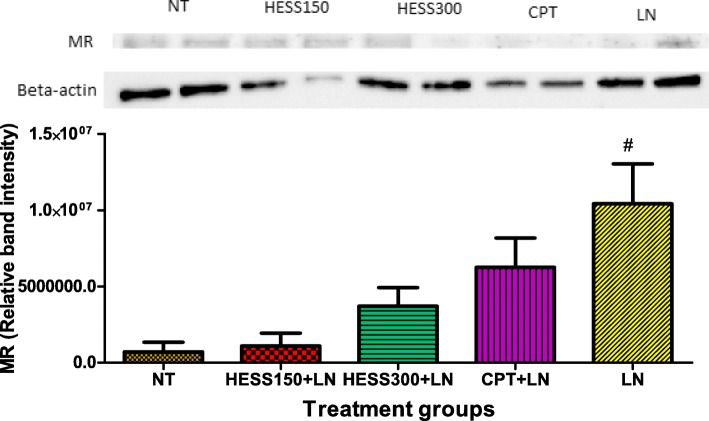
Western blot analysis of mineralocorticoid receptors expression in kidneys of treated rats. The density of each band was evaluated and their ratios to β-actin measured. MR = mineralocorticoid receptor; LN = L-NAME control; CPT + LN = captopril; HESS150 + LN and HESS300 + LN = hydroethanolic extract of *Senecio serratuloides* at 150 and 300 mg/kgrespectively.Results are presented as mean ± SEM. ##*P* < 0.01 compared to NT control group

It was observed that L-NAME treatment significantly increased expression of mineralocorticoid receptors in kidney homogenates of LN group compared to NT group. HESS150 + LN, HESS300 + LN and CPT + LN treatment groups had lower mineralocorticoid receptor levels although there was no significant difference compared to LN group (Fig. 4).

### Nitric oxide concentration in serum, heart and kidney homogenates

**Fig. 5:**
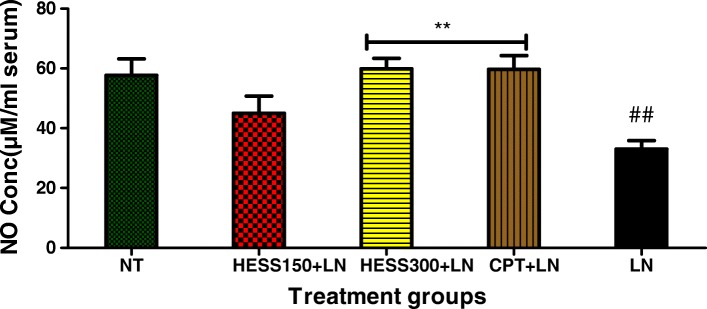
Serum concentration of NO. Values are expressed as mean ± SEM. n = 6; NT = normotensive control; LN = L-NAME control; CPT + LN = captopril; HESS150 + LN and HESS300 + LN = hydroethanolic extract of *Senecio serratuloides* at 150 and 300 mg/kg respectively. **p < 0.01 compared to L-NAME (LN) control group; ##*p* < 0.01 compared to normotensive control group

In serum, L-NAME significantly decreased the concentration of NO in LN group compared to NT group. HESS300 and CPT significantly prevented L-NAME induced decrease in serum NO concentration (Fig. 5).

### Effect of HESS on cardiac tissue

**Fig. 6:**
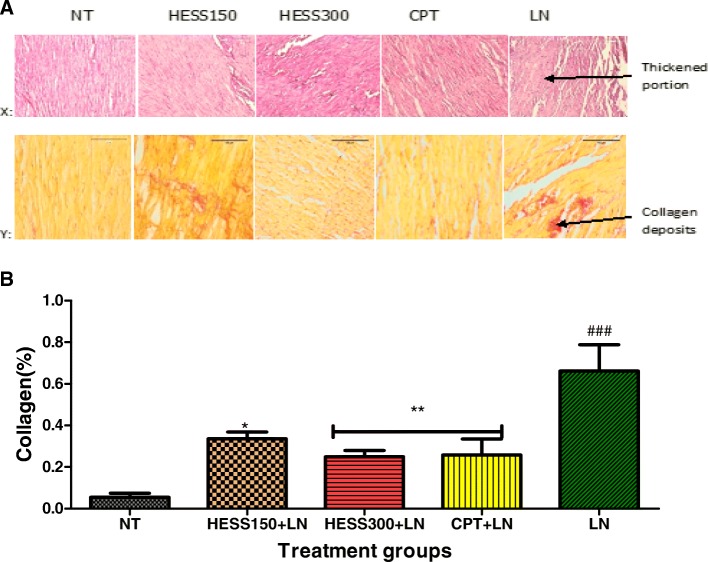
Representative photomicrographs (**a**) of cardiac tissue and graph of semi-quantitative analysis of collagen (**b**). (X)- sections stained with haematoxylin (magnification × 20) and eosin; (Y) - sections stained with picrosirius red stain (magnification × 40); NT- normotensive control; HESS- hydroethanolic extract of *S. serratuloides* at 150 and 300 mg/kg; CPT-captopril group; LN-L-NAME group. Values are expressed as mean ± SEM. *p < 0.05; **P < 0.01 versus LN control; ###*p* < 0.001 versus NT control

Figure 6 shows photomicrographs (A) of cardiac tissue samples stained with haematoxylin and eosin stain (X), picrosirius red stain (Y) and a graph (B) of collagen concentration (%) from semi-quantitative analysis of photomicrographs stained with picrosirius red. L-NAME induced significant fibrosis in LN treatment group compared to NT group (0.66 ± 0.1% vs 0.06 ± 0.02%; *p* < 0.001). On the other hand, L-NAME-induced cardiac fibrosis was attenuated by treatment with HESS150 (0.34 ± 0.02%; 0.05), CPT (0.26 ± 0.1%, 0.01) and HESS300 (0.25 ± 0.03%, 0.01) respectively. Haematoxylin and eosin staining revealed thickened portions of cardiomyocytes in the LN treatment group suggesting hypertrophy that was confirmed with picrosirius stain.

## Discussion

Results from this study showed that the hydroethanolic extract of *S. serratuloides* (HESS) was not toxic and prevented L-NAME induced hypertension. HESS also prevented L-NAME induced hyperlipidemia, maintained serum angiotensin II concentration and protected target organs.

Acute toxicity of HESS was greater than 5000 mg/kg. According to the toxicity guidelines by Konaté et al. [32], pharmacological substances with LD_50_ less than 5 mg/kg are classified as highly toxic substances, those with LD_50_ between 5 mg/kg and 5000 mg/kg are classified as moderately toxic substances while those with LD_50_ > 5000 mg/kg are not toxic. Therefore the hydroethanolic extract of *S. serratuloides* whose LD_50_ > 5000 mg/kg was considered non-toxic and safe for consumption.

Treatment with L-NAME resulted in decreased HDL and increased LDL/TG/VLDL thus corroborating the findings of Salam et al. [33] who showed that L-NAME treatment had an adverse effect on lipid profiles in treated rats. L-NAME treatment raised the concentration of LDL which in turn was capable of interfering with eNOS activity. Under normal conditions, eNOS is associated with cholesterol-enriched caveolae in endothelial cells, where its activity can be carefully regulated [34]. However, in hyperlipidemia, LDL, especially oxLDL negatively affects the activity and sub-cellular distribution of eNOS hence leading to a decrease in NO bioavailability [35, 36]. On the other hand, HDL causes activation of eNOS within the caveolae, with the resultant generation of NO [37]. Our findings showed that HESS significantly improved lipid profiles in a dose dependent manner in L-NAME treated animals indicating that HESS inhibited hypertension by reducing inactivation of eNOS by LDL and promotion of its activity by improved HDL. This was confirmed by its ability to prevent L-NAME induced decrease in serum NO levels. In the heart and kidneys however, the concentration of NO in L-NAME groups was comparable to normotensive control. This finding was consistent with previous studies by Adaramoye et al. [38] and Berkban et al. [39] who also observed normal levels of NO in heart and kidney of L-NAME treated rats. Abdel-Raham et al. [40] proposed that eNOS protein expression may be up-regulated in L-NAME treated rats as a counter-regulatory mechanism to compensate for increased BP. On the other hand Luvarà et al. [41] showed that chronic L-NAME administration was associated with the induction of iNOS expression both at mRNA and protein level. Inducible NOS-derived NO is implicated in the pathogenesis of tissue injury [42] probably through the formation of peroxynitrite and ROS suggesting the pathology observed in cardiac tissue of L-NAME treatment group despite the NO availability. However this finding was inconsistent with Talas et al. [43] who reported decrease in NO concentration in heart and kidney after L-NAME treatment for 15 days. This divergence in findings may be due to differences in duration of studies, dose of L-NAME administered and/or route of administration. In our finding, CPT improved NO availability in the absence of HDL improvement. Similarly, Bernátová et al. [44] showed improved tissue specific NOS activity with CPT at the higher dose of 100 mg/kg, contrary to that reported by Pechanova et al. [45] in which a similar dose of CPT failed to reverse L-NAME induced NO depletion. We have no explanation for this difference at the present moment but it may be worthwhile to investigate CPT effects in a dose response manner.

In this study L-NAME suppressed serum Ang II production thus confirming the finding of Johnson and Freeman [46] who showed that L-NAME decreases serum Ang II concentration. The ability of HESS to maintain serum Ang II at normal levels suggested that it decreased BP by maintaining a balance between Ang II and NO. Endothelial cells generate both Ang II and NO which both have antagonistic effects on vascular smooth muscle cell [47]. Therefore, a balance in concentration of the two molecules is important for maintaining homeostasis.

L-NAME-induced increase in mineralocorticoid receptor concentration in the kidney observed in this study was in line with previous reports that indicated that L-NAME treatment activated the renal renin angiotensin system (RAS) [48] while cardiac hypertrophy was also observed in L-NAME treated rats [49]. These findings suggested the existence of systemic as well as renal RAS thus corroborating studies by Giani et al. [46] which confirmed that L-NAME treated rats are a model of low plasma renin hypertension. These researchers further showed that L-NAME induced a marked activation of the renal RAS in wild-type mice which was absent in the angiotensin converting enzyme (ACE) mutant mice. Angiotensin converting enzyme is the target of ACE inhibitors, which are important medications in the treatment of both L-NAME induced HTN and human essential HTN. The endothelial lining of the lungs is considered the main source of ACE hence it is believed that ACE inhibitors lower BP by inactivating endothelial ACE [50]. However, findings from this study and the fact that many hypertensive patients have normal or low renin levels [51, 52] contradicted this belief. In addition, ACE inhibitors still reduce BP in patients with normal plasma angiotensin II levels [48] further suggesting that the source of ACE may not be limited to the endothelium. Furthermore, besides endothelial ACE, large amounts of ACE are synthesized locally in tissues particularly in the kidney, with potentially broad influences on renal function and ultimately BP [53]. Previous reports by Quiroz et al. [54] and Graciano et al. [48] showed that L-NAME increased renal abundance of several local RAS components, including angiotensinogen, ACE, and the AT1 receptor. Consequently, several authors proposed that intra-renal RAS activation may play a major role in the development of HTN and renal injury, even when there is no clear evidence of increased systemic RAS [46, 55]. This may be due to L-NAME–driven renal inflammation and oxidative stress which can override the physiologic regulation of the local renal RAS and induce its activation [42, 50]. A similar mechanism may explain the effects of L-NAME in cardiac tissue. The role of HESS in treating HTN was further supported by its ability to prevent cardiac hypertrophy and L-NAME induced increase in mineralocorticoid receptor concentration in the kidney in a dose dependent manner. The cardio-protective effect of HESS may have been through inhibition of inflammation and oxidative stress and hence preventing the elevation of RAS components in the heart. Indeed, this plant has been shown to have anti-inflammatory, anti-antioxidant [13] and wound healing effects [14]*.*

## Conclusion

The findings in this study demonstrated that *S. serratuloides* crude extract significantly reduced L-NAME-induced hypertension and L-NAME-induced changes in regulators of blood pressure like nitric oxide and angiotensin II thus showing great therapeutic potential for hypertension.

## Abbreviations

ACE: angiotensin converting enzyme
Ang II: Angiotensin II
AT1: angiotensin receptor type 1
CPT: Captopril (20 mg/kg)
CVD: cardiovascular disease
D_0_: Dose killing no animal
D_100_: Dose killing all animals
DBP: diastolic blood pressure
eNOS: endothelial nitric oxide synthase
HESS: hydroethanol extract of *S. serratuloides*
HESS150: HESS (150 mg/kg)
HESS300: HESS (300 mg/kg)
HRP: horseradish peroxidase
HTN: hypertension
iNOS: inducible nitric oxide synthase
LD_50_: Lethal Dose 50
LN: L-NAME
L-NAME: N-Nitro-L-arginine methyl ester
NEDD: N-(1-naphthyl) ethylenediamine
NO: Nitric oxide
NT: normotensive control
oxLDL: oxidized Low density lipoprotein cholesterol
RAS: renin angiotensin system
ROS: reactive oxygen species
SBP: Systolic blood pressure
TC: HDL + LDL + VLDL
